# Effects of heat treatment on the temperature coefficient of resistivity of laser fabricated NiCr flexible sensors

**DOI:** 10.1186/s40712-026-00430-z

**Published:** 2026-02-26

**Authors:** Hashem Almomani, Jace Menezes, Shivam Abhi, Pablo Enrique, Mohammad Nankali, Peng Peng

**Affiliations:** 1 Department of Mechanical and Mechatronics Engineering, University of Waterloo, Waterloo, ON N2L 3G1 Canada; 2 Centre for Advanced Materials Joining, University of Waterloo, Ontario, N2L 3G1 Canada; 3 Waterloo Institute for Nanotechnology, University of Waterloo, Waterloo, ON N2L 3G1 Canada

**Keywords:** Laser process, Flexible sensors, Heat treatment, Temperature coefficient of resistivity, Thin film alloys

## Abstract

Advancements in data collection and automation require reliable flexible sensors due to their versatility and customization. One challenge with the practical use of flexible sensors is the change in resistance due to the temperature coefficient of resistivity (TCR) at different use temperatures. This research investigates the heat treatment process for a common metal sensing element, Evanohm (80/20 nickel chromium alloy), to reduce its TCR. NiCr thin films were heat treated in an inert argon environment at various temperatures and hold times, then fabricated into flexible sensors on a polymer substrate using UV nanosecond laser ablation. Sensors were mounted onto an aluminum substrate to provide mechanical backing during testing. Following heat treatment, TCR values as low as 1.44 ppm/°C were achieved at a heat treatment temperature of 475°C for 180 min of hold time. Electrical and microstructural changes were also analyzed to determine the mechanism for TCR change. The results demonstrate the effectiveness of heat treatment in reducing the TCR of NiCr flexible sensors, minimizing the impact of use temperature on resistance change. This research contributes to the development of NiCr flexible sensors with stable and linear resistance behaviour over the temperature testing range from -10°C to 150°C.

**Supplementary Information:**

The online version contains supplementary material available at 10.1186/s40712-026-00430-z.

## Introduction

The rapid adoption of artificial intelligence (AI) and business intelligence systems has necessitated the advancement of data collection technologies (McKinsey Global Institute 2017; Thomson and Gresch 2021). Flexible devices, especially sensors, play a pivotal role in enabling precise measurements for automation and decision making (Zhao et al. 2020). These flexible sensors must often withstand harsh operating conditions, including changing use temperature, mechanical stress and electrical interference, while maintaining high accuracy and stability. Nickel chromium (NiCr) alloys, such as Evanohm film, are widely used in resistive strain sensors due to their high resistivity, 85—110 μΩ·cm, and low temperature coefficient of resistivity (TCR), ± 10 PPM/°C (Hamilton Precision Metals 2022). These sensors consist of a thin metal sensing element arranged into a patterned trace and bonded to the substrate. When the substrate is deformed by applied pressure, the deformation translates to the sensing element, leading to a change in resistance that can be measured to calculate the applied force (Zhao et al. 2020; Hoffmann n.d.). In general, flexible resistive sensors are fabricated using thin film deposition techniques such as vapour deposition or sputtering (Zhao et al. 2020; Hoffmann n.d.; Rölke 1981). These techniques produce high quality sensing layers with reliable adhesion to polymer substrates and allow for precise control over thickness and uniformity. Recent studies have also highlighted the growing use of polymer-based sensing systems in flexible functional devices, showing how material selection, geometry and orientation can significantly impact mechanical and thermal performance in various applications, from pipe heating to flexible electronics (Hui et al. 2023; Der et al. 2021; Ordu and Der 2023). Following deposition, additional protective steps, such as lamination or the application of a cover film, are taken to enhance mechanical durability and thermal stability of the sensor during operation (Hoffmann n.d.).

One of the key challenges that flexible resistive sensors face is the increase in resistance at higher operating temperatures. This change in resistance can cause inaccurate results at varying temperature conditions, necessitating the use of other methods, for example compensative circuits, to counterbalance the effect of temperature on resistance (Gray et al. n.d.). Building a cost-effective, easy to integrate, methodology for adjusting the TCR of the sensing element, particularly the NiCr alloys, will reduce the complexity of circuit design, improving the accuracy of fabricated sensors in different temperature environments.

A range of strategies have been explored for TCR control in NiCr alloys. Composition tuning, by adjusting the ratio of alloying elements in the NiCr film, has been shown to be effective (Rölke 1981). Multilayer and nanocomposite structures, such as the introduction of an Al nanolayer in between the NiCr layers, have also been shown to impact resistivity and TCR, with layer thickness providing control over properties (Yu et al. 2022). While these methods can provide precise control, they rely on complex thin film fabrication techniques (Hoffmann n.d.); Rölke 1981; Yu et al. 2022). Although heat treatment could induce grain growth, surface oxidation, and phase segregation in NiCr films, impacting the electrical properties of the sensor (Cheng et al. 2015; Kwon et al. 2005; Reed n.d.), it also provides a simpler method for TCR control of NiCr films pre-sensor fabrication. Previous studies involving the heat treatment of NiCr largely focus on sputtered or vapor deposited thin films, which are then annealed in an inert N_2_ or vacuum environment (Cheng et al. 2015; Kwon et al. 2005; Phuong et al. 2006; Ren et al. 2020; Sun et al. 2019). Under these conditions, heat treatment has been reported to lead to residual stress relaxation, grain growth, and a drop in resistivity, with an observed increase or non-linear TCR change (Cheng et al. 2015; Phuong et al. 2006). In contrast, studies with mechanically processed NiCr films are not as prevalent. This distinction is crucial as rolled and deposited materials may exhibit different surface conditions after heat treatment, leading to a different TCR response in heat treatment.

This research explores the heat treatment process for NiCr film with the aim of reducing the TCR of the material when mounted on an aluminum substrate. Different NiCr films were heat treated between a range of 315 °C and 600 °C for durations of 1—5 h. Their electrical and mechanical properties as well as surface composition were characterized using scanning electron microscopy (SEM), energy dispersive X-ray spectroscopy (EDX) and X-ray photoelectron spectroscopy (XPS). This research aims to increase the customizability of flexible sensors for industrial applications to improve data accuracy under different temperature conditions.

## Methodology

NiCr thin film samples (12 micron thick, Hamilton Precision Metals (2022)) were heat treated in a tube furnace at 315 °C, 475 °C, 500 °C and 600 °C in an inert Ar_2_ atmosphere to inhibit oxidation with a heating rate of 30 °C/min, and a holding time of one hour at the target temperature, followed by gradual cooling to room temperature. Samples were also heat treated at 475 °C for longer hold times to determine the effect of annealing time on TCR stabilization.

This temperature range was selected relative to the melting temperature of the Evanohm (Tm = 1350 °C). Heat treatment at approximately 0.35 of Tm (475°C) was chosen to promote stress relaxation while remaining below 0.5 of Tm to avoid promoting extensive microstructural changes and grain coarsening and is consistent with the annealing temperature for NiCr and related Ni-based alloys (Hamilton Precision Metals 2022; Reed n.d.; Thermal Processing of Metals 2019). 600 °C was included to examine the upper limit of thermal stability, while the lower bound of 315 °C was selected to determine if any TCR change could occur post polyimide lamination (DuPont n.d.), and is the temperature at which the polyimide is cured. The hold time at 475 °C was increased until TCR came close to zero, indicating that resistance is now unaffected by temperature. The nominal composition of Evanohm as received is shown in Table 1.

**Table 1:** The nominal composition of Evanohm as received

Element	% Composition
Chromium	20.0%
Aluminum	2.8%
Copper	2.0%
Silicon	1.0%
Manganese	0.90%
Zirconium	0.08%
Nickel	Balance

**Fig. 1:**
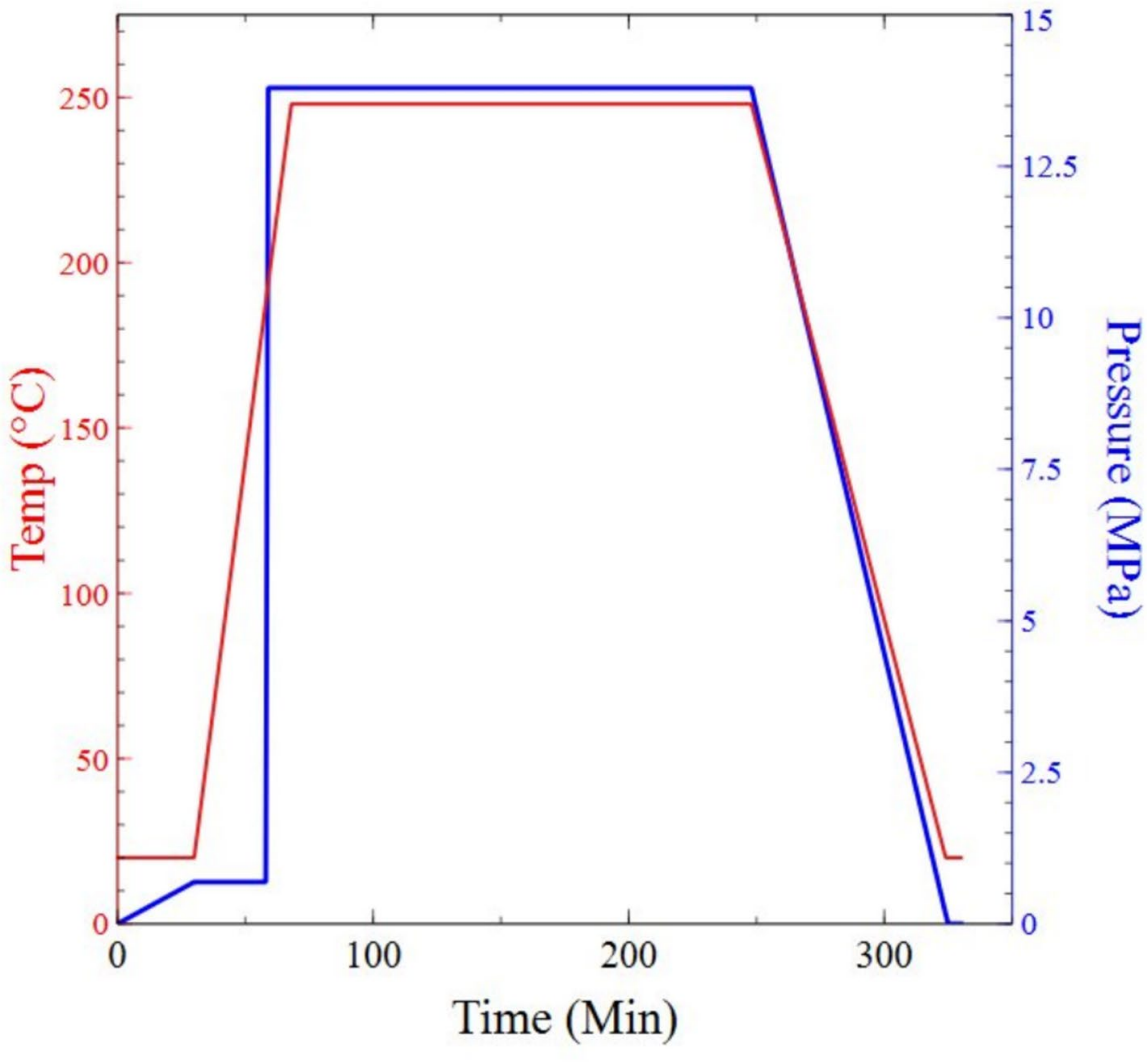
Temperature and pressure vs time for complete polyimide-NiCr-polyimide lamination in hot press

**Fig. 2:**
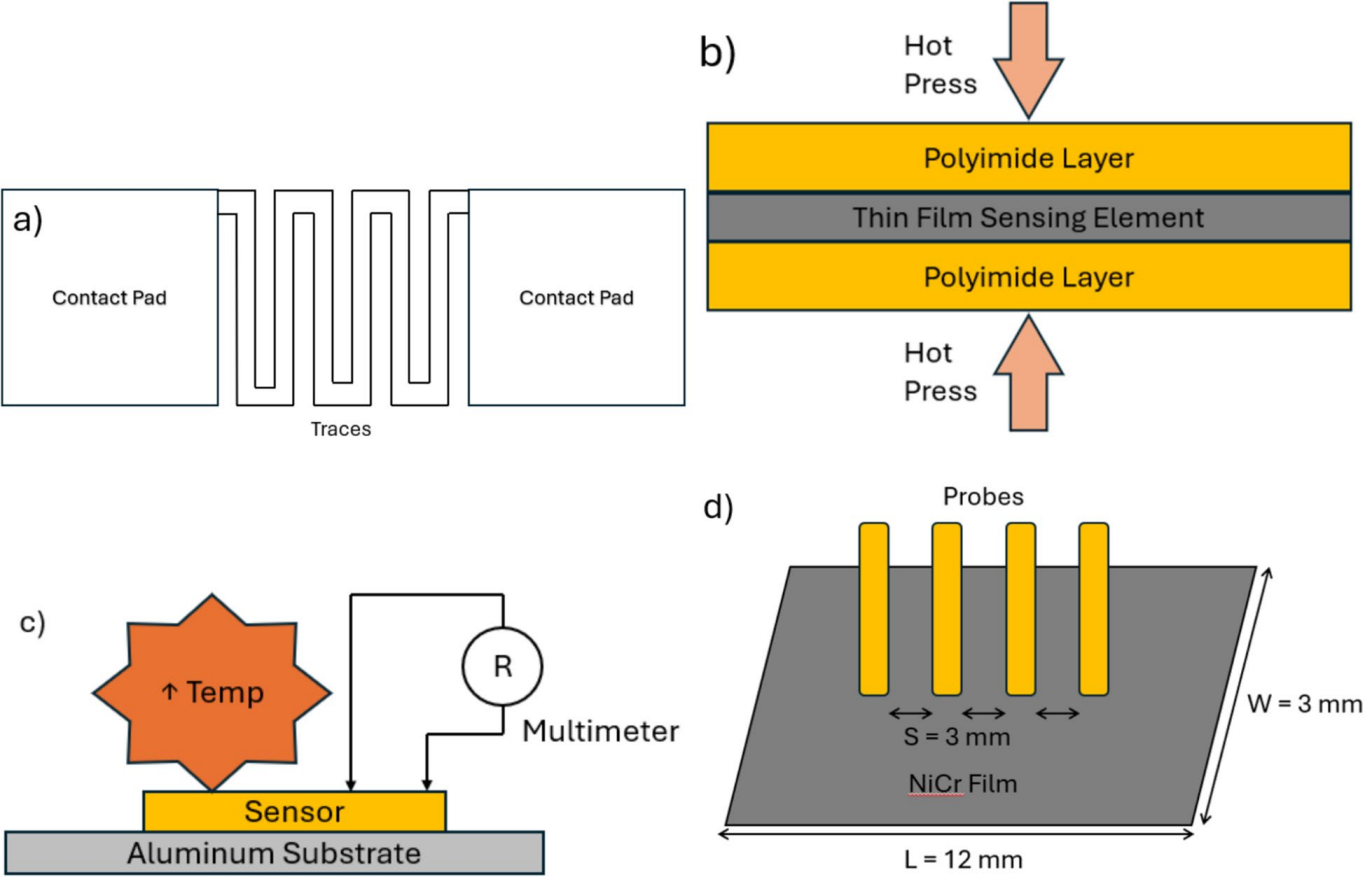
Schematical diagrams of **a**) sample resistive flexible sensor used during TCR testing, **b**) crossectional view of flexbile sensor, and **c**) resistance mearsurement of sensors mounted on alumium substrate at different temperatures, d) 4 point probe set up for resistivity testing with dimensions

To facilitate this TCR testing, resistive flexible sensors that can measure force, torque and deformation (Hoffmann n.d.) will be used. Fig. 2a) provides a sample resistive flexible sensor design for TCR testing of the heat-treated material. The NiCr thin film was hot pressed between two polyimide sheets and cured to provide insulation and backing, as shown in Fig. 2b) (DuPont 2025). The hot-pressing conditions are shown in Fig. 1.

After hot pressing, flexible sensors are then fabricated using pulsed UV nanosecond laser, under previously optimized parameters (Mao et al. 2023), and to minimize any potential effects on the sensing element during fabrication (Leal et al. 2025; Ruffino and Grimaldi 2019). This was done using a diode-pumping solid-state (DPSS) Nd:YVO4 UV pulsed laser (Samurai Desktop 3500). The laser parameters used are shown in Table 2.

**Table 2:** Laser parameters used for sensor fabrication of heat treated and hot-pressed substrate

Parameter	Laser Power (W)	Frequency (kHz)	Mark Speed (mm/s)	# of Passes
Top PI Layer	0.78	42	200	3
NiCr	0.78	38	250	3

Microstructural analysis was performed by examining the surface of the base and heat-treated NiCr thin film using scanning electron microscope (SEM, Zeiss Leo1530 FE-SEM), energy dispersive X-ray at 15 kV acceleration voltage (EDX, Oxford X-Max EDS detector). X-ray photoelectron spectroscopy (XPS) was also conducted using a Nexsa G2 Surface Analysis System and a Thermo-VG Scientific ESCALab 250 microprobes. The obtained XPS analysis was conducted using CasaXPS. The spectra were fitted using a Gaussian–Lorentzian fitting method and a generation of a Shirley background was done using the obtained results Fairley (n.d.). After fitting, the data was extracted, where the background was subtracted from each peak to flatten each curve and provide a better contrast for peak comparison. To validate the effect of heat treatment on bulk material, XRD residual stress analysis was conducted, with an expected drop in residual stress with increasing heat treatment.

After fabrication, the sensors are mounted onto an aluminum substrate for TCR determination, as shown in Fig. 2c. The effectiveness of the heat treatment was evaluated by measuring the resistance of 4 sensors per heat treatment condition across a temperature range of −10°C to 150°C. Once resistance values were obtained, a linear relationship was observed, allowing the TCR to be calculated using Eq. (1) (Ren et al. 2020).

1 $$\boldsymbol T\boldsymbol C\boldsymbol R=\;\frac{{\boldsymbol R}_{\mathbf2}-\;{\boldsymbol R}_{\mathbf1}}{{\boldsymbol R}_{\mathbf1}\left({\boldsymbol T}_{\mathbf2}-\;{\boldsymbol T}_{\mathbf1}\right)}\;\ast\;\mathbf{10}^{\mathbf6}$$

To further validate the changes observed, the heat-treated material was then fabricated into a 3 mm by 12 mm rectangle, with the distance between each probe of 3 mm. This was done to facilitate resistivity testing with a reduced contact-probe resistance, giving a correction factor of 0.2205 (Four-Probe Method: Sheet Resistance Formula Measurement (n.d.); Resistivity Measurements Using the Model 2450 SourceMeter SMU Instrument and a Four-Point Collinear Probe n.d.); Smits 1957). 7 samples of each heat treatment condition were used to determine statistical significance. The schematic is shown in Fig. 2d.

## Results

**Fig. 3:**
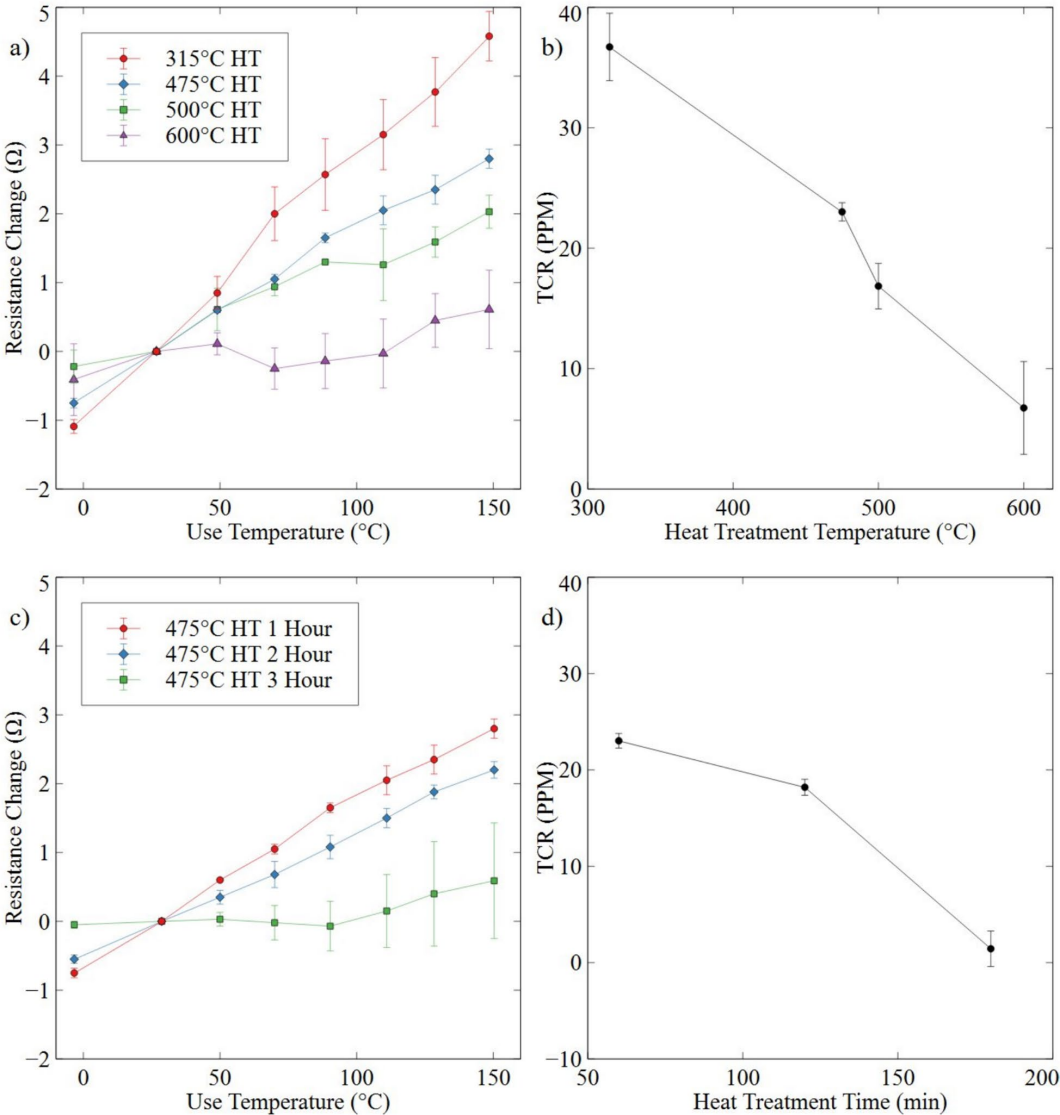
**a**, **c** Resistance of different heat-treated sensors (*n* = 4) measured at various temperatures. Change in TCR with increasing **b** heat treatment temperature and **d** time

Figure 3a shows the resistance changes of the sensors with increasing testing temperature. As heat treatment temperature increases, the resistance changes with increasing testing temperature trends closer to 0. At lower heat treatment temperatures, such as 315 °C, sensors exhibit a larger change in resistance with temperature, reflecting a higher TCR typical of untreated or lightly annealed NiCr thin films (Cheng et al. 2015; Noh et al. 2006). As annealing temperature increases to 475 °C, this change becomes more moderate, suggesting a change in microstructure or surface conditions. At the highest treatment temperature of 600 °C, resistance change becomes minimal, indicating that the change occurring within the microstructure is increasing with heat treatment temperature. This progressive reduction in TCR demonstrates that increasing heat treatment temperatures are effective at reducing the effect of use temperature on resistance within flexible sensors.

This resistance change translates to a reduction of the TCR (Using Eq. 1) as plotted in Fig. 3b. TCR dropping from an average of 36.70 ppm at 315 °C to 5.06 ppm at 600 °C with increasing testing temperature. These results are different from reported effects of annealing temperature on NiCr (Phuong et al. 2006; Ren et al. 2020; Thiel and Maurer 1979), where increasing heat treatment temperature will result in TCR increasing. To determine if the effects observed were similar under different testing conditions, heat treatment was repeated at the optimal annealing temperature of 475 °C for NiCr (Hamilton Precision Metals 2022; Reed n.d. “Thermal Processing of Metals” 2019) but with an increasing holding time. A similar trend was also found, as shown in Fig. 3c and d.

The resistance changes above also translate to a reduction of the TCR with increasing holding time, with TCR dropping from an average of 23.02 ppm at 60 min, to an average of 1.44 ppm at 180 min. Since both increasing temperature and time decrease the TCR, reduced defects and residual stress in bulk NiCr by annealing (Phuong et al. 2006; Ren et al. 2020; Thiel and Maurer 1979) may not be the contributing factors in this case. Usually, when annealed in an inert atmosphere (nitrogen, argon, etc.) the TCR of NiCr alloys is increased as defect reduction allows for more homogenous electron transport throughout the material (Ren et al. 2020). In this study, a competing mechanism may dominate the TCR change, requiring more characterization. To validate if bulk changes are occurring with the current heat treatment conditions, XRD was conducted to determine the residual stress of each sample. Table 3 shows a drop in residual stress with increasing heat treatment temperature. In prior studies, this reduction correlates to an increase in TCR, primarily driven by grain growth induced during heat treatment (Sun et al. 2019).

**Table 3:** Heat Treatment conditions vs residual stress values in MPa

Sample	Residual Stress value (MPa)
Base Metal	−1091.3
315 °C	−632.3
475 °C	−414.6
600 °C	−12.3

Figure 3 shows the surface change of the NiCr with increasing heat treatment temperature and time. The surface irregularities increase with increasing heat treatment, highlighted by an increasing porosity and a build up of surface clusters. To better understand the surface composition of these changes, EDX was conducted. The resulting EDX data showing the atomic percentage of elements on the whole surface at each heat treatment temperature is shown in Table 4, providing a basis for what elements of interest should be evaluated using XPS for more surface condition specific analysis.

**Table 4:** EDX composition of heat treated samples

	No HT	425°C	475 °C 3 Hours	600 °C 5 h
Element	at. %	at. %	at. %	at. %
O				16.2
Si			1.9	1.8
Cr	21.2	22.8	21.5	18.4
Ni	70.9	69.6	69.2	56.9
Trace (Al, Mn)	Balance	Balance	Balance	Balance

The above reveals a steady decrease in Ni atomic percentage at higher heat treatment temperatures, correlating with the increase in O atomic percentage, indicating the formation of an oxide at the surface. Si also increased with increasing heat treatment time and temperature. This redistribution reflects thermally driven surface migration of elements along with surface oxidation, both common with heat treatment processes (Cheng et al. 2015; Phuong et al. 2006; Zhou et al. 2003). Above 425 °C, Si, the only element known to exhibit a negative TCR of −0.04 ppm (Cheng et al. 2015), migrates to the surface. Its accumulation at the surface suppresses the expected increase in TCR, counteracting the typical behaviour observed during heat treatment and annealing (Phuong et al. 2006; Ren et al. 2020; Thiel and Maurer 1979).

**Fig. 4:**
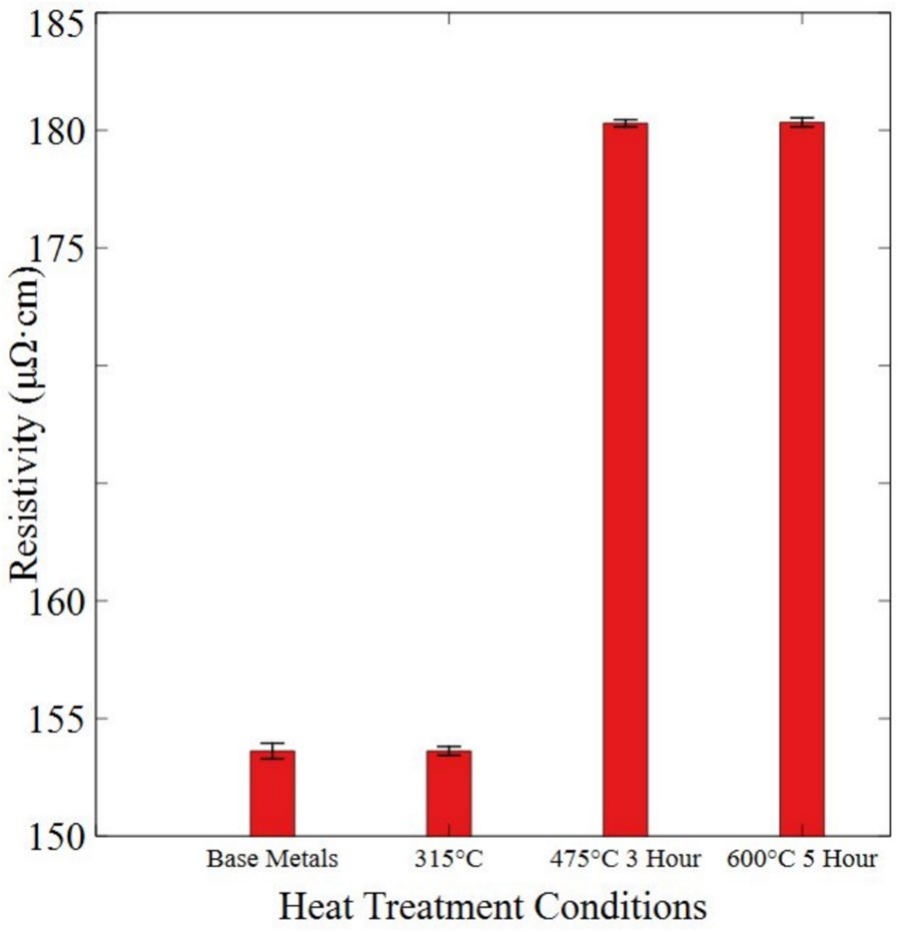
Resistivity changes of sensors (*n* = 7) at different heat treatment conditions

**Fig. 5:**
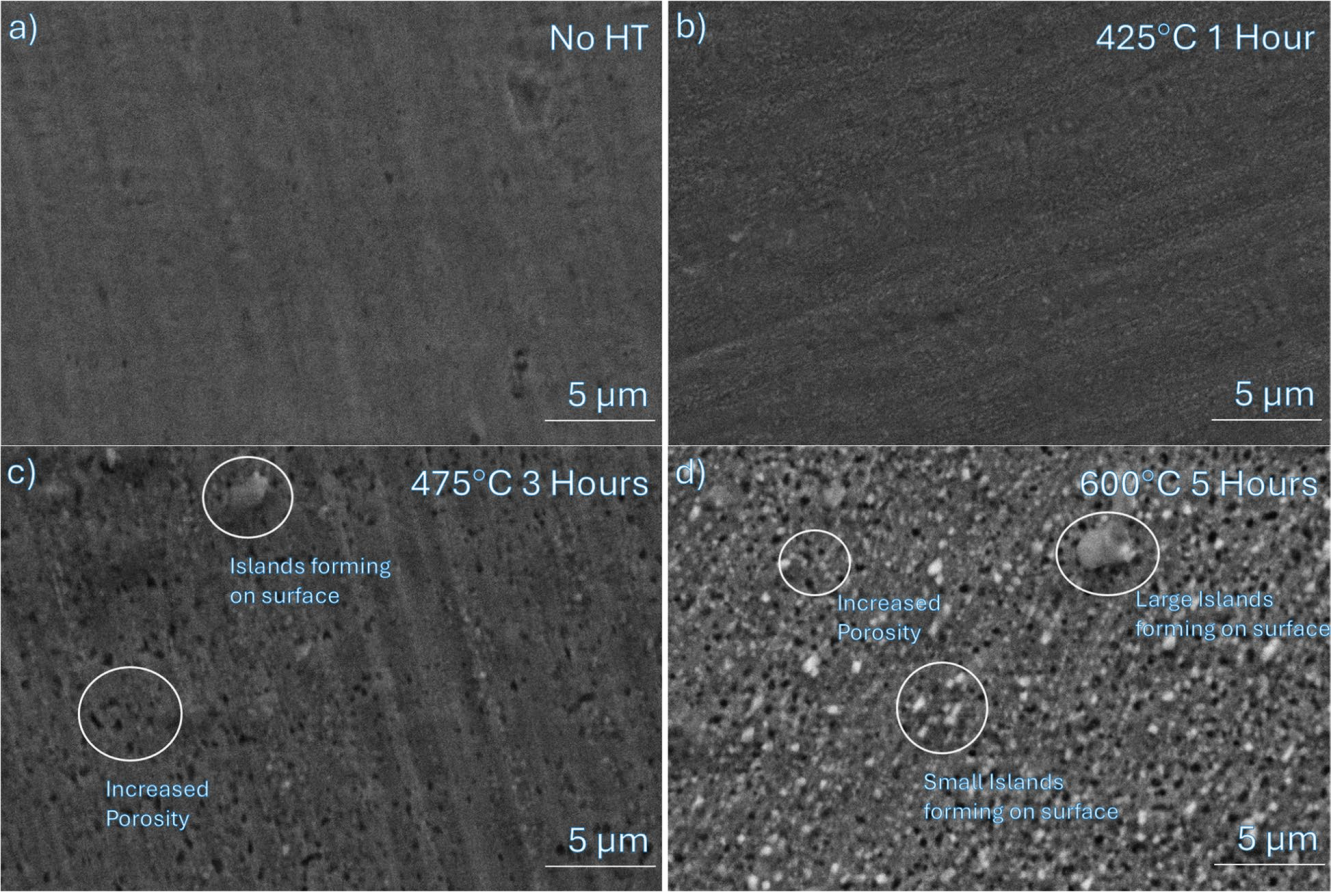
SEM image of different heat treated sensors: **a** as rolled material **b** 425 °C for 1 h **c** 475 °C for 3 h **d**) 600 °C for 5 h

To validate the increased existence of oxides on the surface, a 4-point probe test was conducted to determine the resistivity of samples at different heat treatment conditions, as shown in Fig. 4. The resistivity of the NiCr at higher heat treatment temperature and longer time increases from 154 (μΩ·cm) to 180 (μΩ·cm). This increase can be attributed to the increased surface oxidation observed in the EDX results, more specifically the formation of nickel oxides (Noh et al. 2006; Lee et al. 2011). The increased irregularity on the surface, evidenced in clusters and pores, as observed in Fig. 5c and d, creates localized disruption at the surface, acting as scattering centers for electron transport (Ren et al. 2020), contributing to the TCR drop. This is further supported by the increased resistivity observed, as resistivity and TCR are negatively correlated, meaning that in increase in resistivity leads to a decrease in TCR (Hill et al. 2013).

**Fig. 6:**
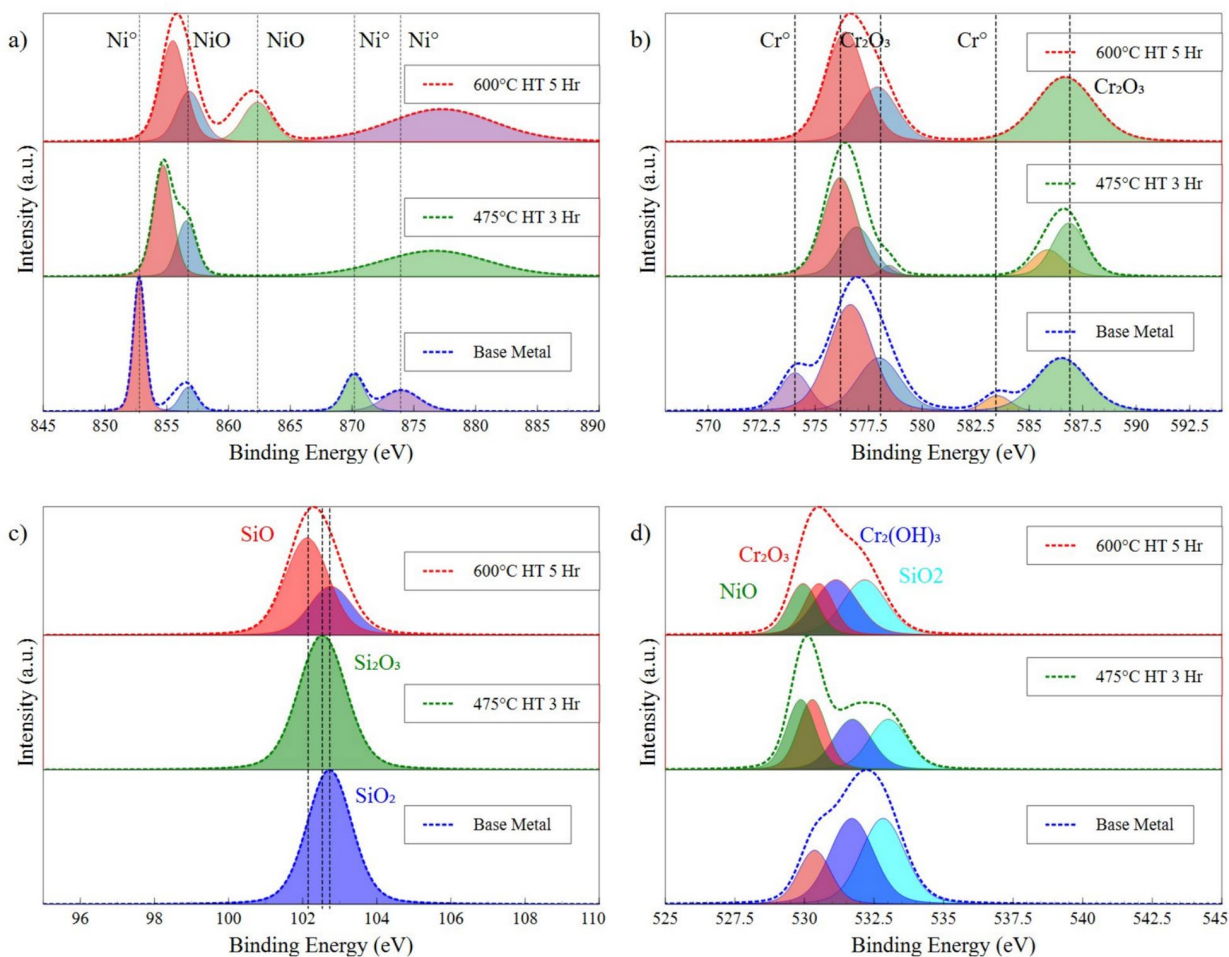
XPS spectra with increasing HT temperature. **a** Ni 2p spectra, **b** Cr 2p spectra, **c** Si 2p spectra, **d** O 1 s spectra

To further investigate the chemical changes of surface conditions, XPS was conducted. Figure 6 provides a comparison of the spectra for the base metal, heat treated samples at 475 °C for 3 h and 600 °C for 5 h. The Ni 2p spectrum of the base metal in Fig. 6a exhibits characteristic metallic nickel peaks at 852.8 eV (2p₃/₂) and 870.0 eV (2p₁/₂) (Furstenau et al. 1985; Hsu and Williams 1994), with some satellite features at 856.6 eV and 874.2 eV (Mansour 1994; Mclntyre et al. n.d.), most commonly associated with surface oxidation or partial Ni^2^⁺. After heat treatment at 475 °C, the metallic peaks begin to shift right and the dominant feature at 856.0 eV reflects increased oxidation to Ni^2^⁺, consistent with NiO formation Mclntyre et al. (n.d.). At 600 °C, a new peak emerges at 862.2 eV (Bianchi et al. 1993), typically attributed to disordered or mixed Ni oxides, suggesting that the surface is becoming chemically more complex and heavily oxidized. These transformations are strongly correlated with the increased resistivity and drop in TCR as NiO formation disrupts metallic conduction pathways and reduces temperature sensitivity (Noh et al. 2006; Lee et al. 2011). The Cr 2p spectra remain centered at 576.8 eV and 587.1 eV across all samples, consistent with Cr^3^⁺ in Cr₂O₃, a known passivating oxide in nichrome films (Zhou et al. 2003; Kemnitz et al. 1996; Ikemoto et al. n.d.). However, the base metal exhibits additional satellite peaks at 574.1 eV and 586.8 eV associated with metallic Cr or sub-oxides (Sleigh et al. 1996; Desimoni et al. 1988) as shown in Fig. 6b. The disappearance of these satellite peaks following heat treatment suggests that Cr undergoes full oxidation and stabilizes as Cr₂O₃, which remains largely inert during further heating. This supports the interpretation that Cr₂O₃ acts as a chemically stable but passive layer that is gradually overlaid by the more reactive NiO layer at higher temperatures.

The Si 2p spectra in Fig. 6c shifts progressively left, from 102.7 eV in the base metal, to 102.5 eV at 475 °C and 102.1 eV at 600 °C, with the 102.7 eV peak reappearing again. These peaks are associated with SiO_2_, Si_2_O_3_, and SiO binding (Kaur et al. 2016; González-Elipe et al. 1991; Alfonsetti et al. 1993), with the decrease reflecting a transition from a higher to lower Si:O ratio with increasing heat treatment, as oxygen begins to bind with Ni on the surface (Alfonsetti et al. 1993; Battistoni et al. 1981). The formation of these reduced SiO_x_ species suggests a reduction of Si with increasing heat treatment temperature, leading to the development of mixed oxides and defects on the surface (Cheng et al. 2015; González-Elipe et al. 1991; Alfonsetti et al. 1993). The O 1 s spectra in Fig. 6d provide critical evidence for these oxidation processes. With a shift left indicating a growing increase of metallic oxides (Lo et al. 1995; Dickinson et al. 1976). In the base metal, the dominant spectra are centered around 531.7 eV, most closely associated with Cr_2_(OH)_3_ and SiO_2_ (Preparation of metal-polymer dispersions by plasma techniques n.d.); Finster et al. n.d. De-convulsion reveals additional spectra indicating the existence of Cr_2_O_3_ (Sleigh et al. 1996), correlating with the smaller observed Cr^3+^ peaks in the Cr 2p spectra. With increasing heat treatment, the observed binding energy shifts more to the left, with the reduction indicating an increase in NiO correlated peaks (Lo et al. 1995; Marcus and Grimal 1992). Increasing heat treatment to 600 °C maintains this shift, while broadening the spectra, denoting the chaotic and disorganized oxides that are forming on the surface, both Cr_2_O_3_ and NiO (Dickinson et al. 1976; Preparation of metal-polymer dispersions by plasma techniques n.d.; Marcus and Grimal 1992; Pit’Ts n.d.). 

The area under each characteristic curve was calculated to compare compositional changes on the surface, as shown in Table 5. For Ni, all of the originally metallic contribution present in the base metal is completely replaced by NiO after heat treatment. This confirms that heat treatment promotes the enrichment of Ni based oxides at the surface, which become the major surface phase, dominating the XPS response (Mansour 1994; Bianchi et al. 1993). Similarly, the Cr 2p shows a marked reduction of the percentage of metallic Cr. After heat treatment, the primary surface specimen is Cr₂O₃, reaffirming its role as the initial passivating oxide (Zhou et al. 2003). The Si 2p spectrum does not change significantly between specimen, but shifts to lower binding energy at high temperature with an obvious SiO peak at 102.2 eV (Alfonsetti et al. 1993), aligning with the expected formation of small SiO islands on the surface (Cheng et al. 2015) as shown in the SEM images in Fig. 3. The calculated compositional change of the O 1 s spectra shows the clearest change in surface conditions. In the base film, oxygen is dominated by Cr_2_(OH)_3_ and SiO₂ contributions (Preparation of metal-polymer dispersions by plasma techniques (n.d.); Finster et al. (n.d.) Kim and Winograd 1974), while at 475 °C the metal oxide percentage begins to increase, reflecting the growth of NiO and Cr₂O₃ (Lo et al. 1995; Dickinson et al. 1976). This increase persists at 600 °C, with the observed spectra widening. This widening is attributed to defect rich or chaotic oxide environments, consistent with the pores and small island morphologies observed in SEM in Fig. 5d.

**Table 5:** Calculated percentages of each compound from XPS spectra

Ni 2p					
Base Metal		475 °C 3 Hours		600 °C 5 Hours	
Ni	88%	Ni	0%	Ni	0%
NiO	12%	NiO	100%	NiO	100%

Cr 2p					
Base Metal		475 °C		600°C	
Cr	35%	Cr	0%	Cr	0%
Cr_2_O_3_	65%	Cr_2_O_3_	98%	Cr_2_O_3_	100%
CrO_3_	0%	CrO_3_	2%	CrO_3_	0%

O 1 s					
Base Metal		475°C		600°C	
Cr_2_O_3_	19%	Cr_2_O_3_	25%	Cr_2_O_3_	19%
NiO	0%	NiO	25%	NiO	19%
Cr_2_(OH)_3_	40%	Cr_2_(OH)_3_	25%	Cr_2_(OH)_3_	31%
SiO_2_	41%	SiO_2_	25%	SiO_2_	31%

The resistance change was then remeasured for the same specimen after 8 months of storage. Samples were cycled from 25 °C to 150 °C three times, and the resistance change was remeasured across the original temperature range. The difference between the original results vs the remeasured results was graphed vs the −10°C to 150 °C use temperature to determine sensor stability. The resulting variability in resistance measured below 1 Ω across all heat treatment conditions and temperatures.

## Discussion

The SEM and XPS results reveal that heat treatment leads to the formation of a heterogeneous, NiO-rich oxide layer on the NiCr surface, potentially altering the presence of the Cr₂O₃ passivating layer. These surface changes are consistent with the observed decline in TCR, suggesting a shift from metallic conduction to mixed conduction dominated by more oxide interfaces (Noh et al. 2006; Lee et al. 2011). This behaviour likely reflects additional contributions to TCR change beyond stress relaxation and grain growth that are typically associated with bulk heat treatment (Sun et al. 2019).

Although the oxide layer formed during heat treatment is expected to be thin compared to the 12 μm NiCr film, its growth can significantly impact the electrical transport mechanism by modifying the conduction pathways (Mayadas and Shatzkes 1970).The formation of a resistive surface oxide reduces the metallic cross-section and introduces a metal-oxide interface that acts as electron scattering sites (Noh et al. 2006; Mayadas and Shatzkes 1970; Au et al. 1987). As oxide coverage and interfacial roughness increase with heat treatment conditions, resistivity also increases, resulting in a decrease in TCR (Yu et al. 2022; Cheng et al. 2015; Phuong et al. 2006; Noh et al. 2006). This effective modification of conduction pathways provides an explanation for the observed decrease in TCR.

At 475 °C heat treatment temperature, precipitates within the NiCr matrix have been reported to coarsen (Reed (n.d.), and promote grain growth (“Thermal Processing of Metals” 2019). The observed decrease in TCR in heat treated samples is different from previously studied deposited NiCr films (Ren et al. 2020). In previous studies, TCR increases with heat treatment are often attributed to an increase in grain size and reduction of defects, allowing the material to behave more metallic (Phuong et al. 2006). As grains grow, there are fewer grain boundaries within the material to scatter electrons, reducing the resistivity of the material (Thiel and Maurer 1979), and in turn increasing the TCR of the material with increased heat treatment temperature and time. In this study, XRD residual stress measurements show a reduction in stress with increasing heat treatment temperature, consistent with observed microstructural relaxation that occurs during heat treatment (Sun et al. 2019), but does not account for the observed decrease in TCR.

The discrepancy in the observed TCR behaviour in this study is likely due to the surface conditions and compositional change of the heat-treated NiCr at higher temperature or longer hold times. The NiCr film contains trace elements (e.g., Si) that redistribute during heat treatment, introducing a competing mechanism of TCR control, seemingly overriding the expected effect of grain growth. As shown in Fig. 5, increasing heat treatment temperature and time promotes the migration of Si, Ni, and Cr to the surface, adding irregularity and promoting defect formation. EDX results in Table 4 show the increasing atomic percentage of these elements at the surface across different heat treatment conditions, supporting the role of elemental redistribution in modifying surface conditions. These defects and secondary phases form islands, shown in Fig. 5, which could dominate the TCR effect of heat treatment, leading to a drop in TCR. For example, increased surface oxides would have a near 0 or negative TCR (Cheng et al. 2015; Au et al. 1987), as XPS results suggested NiO at the surface increased with increasing heat treatment conditions, both temperature and hold time at 475°C. The formation of islands, along with defects present as pores and clusters, allows for additional scattering centers for electrons, reducing TCR (Au et al. 1987), and can inhibit grain growth, reducing the propensity for the expected TCR increase with heat treatment (Ren et al. 2020).

To summarize, the TCR decrease observed in this study for aluminum-mounted NiCr sensors is primarily driven by surface conditions changing during heat treatment, as opposed to bulk microstructural changes alone. Heat treatment promotes the surface migration of Ni and other trace elements, resulting in the formation of secondary phases, defects, and an oxide-rich surface composition. These features exhibit a near-zero or negative TCR behaviour and suppress the positive TCR contribution typically associated with heat-treatment induced grain growth and stress relaxation, allowing surface conditions to dominate the TCR response. Further surface characterization is needed to quantify the compounds formed and to better resolve the mechanisms for TCR reduction. Extending this approach to NiCr sensors mounted on other materials besides aluminum will be important for isolating interfacial contributions to any measured resistance change, allowing for more TCR control when mounted on other substrates for future applications.

## Conclusion

The observed reduction in the temperature coefficient of resistance (TCR) in heat-treated, aluminum-mounted NiCr thin film sensors highlights the interplay between surface chemistry, elemental migration and oxide formation. At lower heat treatment temperatures, the TCR was measured to be 36.70 ppm at 315 °C and decreased to 5.06 ppm following heat treatment at 600°C. Increasing heat treatment hold time to 180 min at 475 °C further reduces the TCR to 1.44 ppm. The formation of NiO, Cr₂O₃, along with the accumulation of SiO-rich islands on the surface, introduces more electron-scattering centers and disrupts metallic conduction pathways to reduce the sensitivity of the NiCr sensing element to temperature. These findings support a model in which heat treatment not only alters film microstructure but also adjusts TCR through surface oxidation and trace element surface migration. This work demonstrates a material level approach for reducing the TCR of aluminum-mounted flexible NiCr sensors to as low as 1.44 ppm through controlled heat treatment at 475 °C for 180 min. As heat treatment is done at the a material level and before polymer lamination, potential thermal degradation of polymer substrates is avoided, allowing this process to be integrated into existing flexible sensor fabrication workflows.

## Supplementary Information

Supplementary Material 1.

## Data Availability

All data collected for this study is presented in the tables and figures.
